# Ethyl 5-amino-3-(pyridin-4-yl)-1-(2,4,6-tri­chloro­phen­yl)-1*H*-pyrazole-4-carb­oxyl­ate dimethyl sulfoxide hemisolvate

**DOI:** 10.1107/S1600536812008264

**Published:** 2012-02-29

**Authors:** Bassam Abu Thaher, Pierre Koch, Dieter Schollmeyer, Stefan Laufer

**Affiliations:** a Faculty of Science, Chemistry Department, Islamic University of Gaza, Gaza Strip, Palestinian Territories; b Institute of Pharmacy, Department of Pharmaceutical and Medicinal Chemistry, Eberhard Karls University Tübingen, Auf der Morgenstelle 8, 72076 Tübingen, Germany; c Department of Organic Chemistry, Johannes Gutenberg-University Mainz, Duesbergweg 10-14, D-55099 Mainz, Germany

## Abstract

The asymmetric unit of the title compound, C_17_H_13_Cl_3_N_4_O_2_·0.5C_2_H_6_OS, contains two almost identical mol­ecules and one dimethyl sulfoxide (DMSO-*d*_6_) solvent mol­ecule. The pyrazole ring forms dihedral angles of 54.6 (4) and 80.0 (4)° in one mol­ecule, and dihedral angles of 54.2 (4) and 81.2 (4)° in the other mol­ecule, with the directly attached pyridine and trichloro­phenyl rings, respectively. The dihedral angles of the pyridine and trichloro­phenyl rings are 51.2 (4) and 52.0 (4)°, respectively. The crystal packing is characterized by intra- and inter­molecular hydrogen bonds. The crystal is a nonmerohedral twin with a contribution of 0.488 (1) for the minor twin component. The terminal ethyl group of one mol­ecule and the S atom of DMSO are disordered over two sites.

## Related literature

For pyridinyl-substituted five-membered heterocycles as p38α MAP kinase inhibitors, see: Abu Thaher *et al.* (2009 ▶); Peifer *et al.* (2006 ▶). For inhibitory activity and preparation of the title compound, see: Abu Thaher, Arnsmann *et al.* (2012 ▶). For related structures, see: Abu Thaher, Koch *et al.* (2012*a* ▶,*b* ▶).
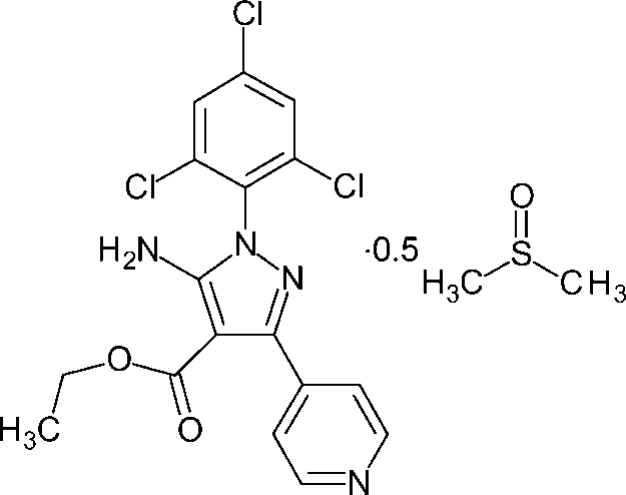


## Experimental

### 

#### Crystal data

C_17_H_13_Cl_3_N_4_O_2_·0.5C_2_H_6_OS
*M* *_r_* = 450.73
Monoclinic, 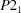

*a* = 13.501 (2) Å
*b* = 10.3222 (15) Å
*c* = 14.889 (2) Å
β = 100.453 (5)°
*V* = 2040.5 (5) Å^3^
*Z* = 4
Mo *K*α radiation
μ = 0.53 mm^−1^
*T* = 173 K
0.40 × 0.20 × 0.05 mm

#### Data collection

Bruker APEXII diffractometer
Absorption correction: multi-scan (*TWINABS*; Sheldrick, 2008*b* ▶) *T*_min_ = 0.818, *T*_max_ = 0.974
9562 measured reflections
9562 independent reflections
5575 reflections with *I* > 2σ(*I*)

#### Refinement

*R*[*F* ^2^ > 2σ(*F* ^2^)] = 0.068
*wR*(*F* ^2^) = 0.133
*S* = 0.98
9562 reflections
526 parameters
19 restraints
H-atom parameters constrained
Δρ_max_ = 0.39 e Å^−3^
Δρ_min_ = −0.39 e Å^−3^
Absolute structure: Flack (1983 ▶), 4289 Friedel pairs
Flack parameter: 0.61 (10)

### 

Data collection: *APEX2* (Bruker, 2006 ▶); cell refinement: *SAINT* (Bruker, 2006 ▶); data reduction: *SAINT*; program(s) used to solve structure: *SIR97* (Altomare *et al.*, 1999 ▶); program(s) used to refine structure: *SHELXL97* (Sheldrick, 2008*a* ▶); molecular graphics: *PLATON* (Spek, 2009 ▶); software used to prepare material for publication: *PLATON*.

## Supplementary Material

Crystal structure: contains datablock(s) I, global. DOI: 10.1107/S1600536812008264/bt5829sup1.cif

Structure factors: contains datablock(s) I. DOI: 10.1107/S1600536812008264/bt5829Isup2.hkl

Supplementary material file. DOI: 10.1107/S1600536812008264/bt5829Isup3.cml

Additional supplementary materials:  crystallographic information; 3D view; checkCIF report

## Figures and Tables

**Table 1:** Hydrogen-bond geometry (Å, °)

*D*—H⋯*A*	*D*—H	H⋯*A*	*D*⋯*A*	*D*—H⋯*A*
N12*A*—H12*A*⋯O15*A*	0.88	2.34	2.767 (8)	110
N12*A*—H12*A*⋯O14*B*^i^	0.88	2.10	2.957 (8)	164
N12*A*—H12*B*⋯N21*A*^ii^	0.94	2.15	2.906 (9)	137
N12*B*—H12*C*⋯O15*B*	0.88	2.13	2.778 (8)	130
N12*B*—H12*C*⋯O14*A*^iii^	0.88	2.24	2.983 (8)	142
N12*B*—H12*D*⋯N21*B*^ii^	0.88	2.02	2.901 (9)	176

Symmetry codes: (i) 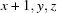; (ii) 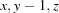; (iii) 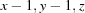.

**Fig. 1.**
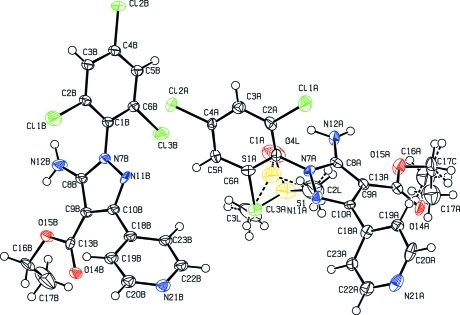
View of the title compound. Displacement ellipsoids are drawn at the 50% probability level. H atoms are depicted as circles of arbitrary size.

**Fig. 2.**
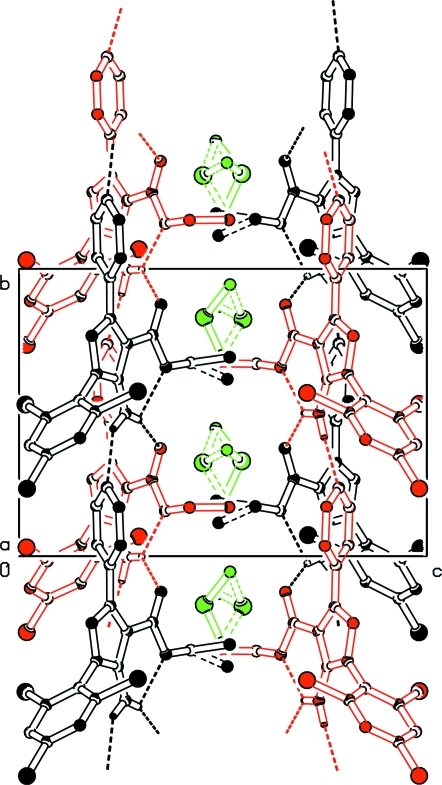
Crystal structure of the title compound with view along the a-axis. Hydrogen bonding is shown with dashed lines. Molecule A coloured in black, B in red and DMSO in green.

## supplementary crystallographic information

### Comment

Pyridin-4-yl substituted five-membered heterocycles have been considered to be potential p38α MAP kinase inhibitors (Abu Thaher *et al.* 2009; Peifer *et al.* 2006). We showed that the regioisomeric switch from 3-(4-fluorophenyl)-4-(pyridin-4-yl)-1-(aryl)-1*H*-pyrazol-5-amine to 4-(4-fluorophenyl)-3-(pyridin-4-yl)-1-(aryl)-1*H*-pyrazol-5-amine completely changed the inhibitory profile from p38α MAP kinase to kinases releant in cancer (Abu Thaher, Arnsmann *et al.* 2012). Recently, we reported similar crystal structures (Abu Thaher, Koch *et al.* 2012*a*,*b*).

The asymmetric unit of the title compound contains two almost identical molecules and DMSO-*d*6 (Fig. 1). In molecule A the pyrazole ring forms dihedral angles of 54.6 (4)° and 80.0 (4)° with the directly attached pyridine and trichlorophenyl rings, respectively. In molecule B the pyrazole ring forms dihedral angels of 54.2 (4)° and 81.2 (4)° with the pyridine and trichlorophenyl rings, respectively. The dihedral angle of the pyridine and trichlorophenyl rings in molecules A and B are 51.1 (4)° and 52.0 (4)°, respectively.

The crystal packing (Fig. 2) shows that the amino function (N12) acts as a hydrogen bond donor forming an intramolecular hydrogen bond to the oxygen atom (O15) and two intermolecular hydrogen bonds to the nitrogen atom of the pyridine ring (N21) (A—A/B—B) and to the carbonyl oxygen atom (O14) of the ester moiety of two different molecules (A—B/B—A) resulting in a double chain along the *b* axis.

### Experimental

4 mmol of *N*-(2,4,6-trichlorophenyl)-4-pyridinecarbohydrazonoyl chloride and 1.5 equiv. of ethyl cyanoacetate were dissolved in 20 ml dry ethanol and cooled to 273 K in an ice bath. 2.0 equiv. of sodium ethoxide solution (21% ethanol) was added dropwise and the reaction was stirred over night. The precipitate was filtered from the reaction mixture, washed with water and recrystallized from hot ethanol. Yield: 56%. Suitable crystals for X-ray were taken from the NMR-tube in DMSO-*d_6_*.

### Refinement

Hydrogen atoms attached to carbons were placed at calculated positions with C—H = 0.95 Å (aromatic) or 0.98–0.99 Å (*sp*^3^ C-atom). All H atoms were refined in the riding-model approximation with isotropic displacement parameters (set at 1.2–1.5 times of the *U*_eq_ of the parent atom). Obtained crystals were twinned. Using the twin matrix -1 0 0, 0 - 1 0, -.4 0 1 with BSAF 0.488 (2) the structure refinement was successful. The solvent molecule and the ethyl group are disorderd with site occupation factors of 0.55 and 0.737 (6) for the major occupied site. The displacement ellipsoids of the atoms C17A, C17C and C2B were restrained to an isotropic behaviour with an effective esd of 0.01 for C17A and C17C and 0.005 for C2B.

### Crystal data

**Table:**

C_17_H_13_Cl_3_N_4_O_2_·0.5C_2_H_6_OS	*F*(000) = 924
*M**_r_* = 450.73	*D*_x_ = 1.467 Mg m^−^^3^
Monoclinic, *P*2_1_	Mo *K*α radiation, λ = 0.71069 Å
Hall symbol: P 2yb	Cell parameters from 3021 reflections
*a* = 13.501 (2) Å	θ = 2.2–23.0°
*b* = 10.3222 (15) Å	µ = 0.53 mm^−^^1^
*c* = 14.889 (2) Å	*T* = 173 K
β = 100.453 (5)°	Plate, colourless
*V* = 2040.5 (5) Å^3^	0.40 × 0.20 × 0.05 mm
*Z* = 4	

### Data collection

**Table:**

Bruker APEXII diffractometer	9562 independent reflections
Radiation source: sealed tube	5575 reflections with *I* > 2σ(*I*)
Graphite monochromator	*R*_int_ = 0.000
CCD scan	θ_max_ = 27.9°, θ_min_ = 1.9°
Absorption correction: multi-scan (TWINABS; Sheldrick, 2008*b*)	*h* = −17→17
*T*_min_ = 0.818, *T*_max_ = 0.974	*k* = −13→13
9562 measured reflections	*l* = 0→19

### Refinement

**Table:**

Refinement on *F*^2^	Secondary atom site location: difference Fourier map
Least-squares matrix: full	Hydrogen site location: inferred from neighbouring sites
*R*[*F*^2^ > 2σ(*F*^2^)] = 0.068	H-atom parameters constrained
*wR*(*F*^2^) = 0.133	*w* = 1/[σ^2^(*F*_o_^2^) + (0.0523*P*)^2^] where *P* = (*F*_o_^2^ + 2*F*_c_^2^)/3
*S* = 0.98	(Δ/σ)_max_ < 0.001
9562 reflections	Δρ_max_ = 0.39 e Å^−^^3^
526 parameters	Δρ_min_ = −0.39 e Å^−^^3^
19 restraints	Absolute structure: Flack (1983), 4289 Friedel pairs
Primary atom site location: structure-invariant direct methods	Flack parameter: 0.61 (10)

### Special details

**Table:**

Geometry. All e.s.d.'s (except the e.s.d. in the dihedral angle between two l.s. planes) are estimated using the full covariance matrix. The cell e.s.d.'s are taken into account individually in the estimation of e.s.d.'s in distances, angles and torsion angles; correlations between e.s.d.'s in cell parameters are only used when they are defined by crystal symmetry. An approximate (isotropic) treatment of cell e.s.d.'s is used for estimating e.s.d.'s involving l.s. planes.
Refinement. Refinement of *F*^2^ against ALL reflections. The weighted *R*-factor *wR* and goodness of fit *S* are based on *F*^2^, conventional *R*-factors *R* are based on *F*, with *F* set to zero for negative *F*^2^. The threshold expression of *F*^2^ > σ(*F*^2^) is used only for calculating *R*-factors(gt) *etc*. and is not relevant to the choice of reflections for refinement. *R*-factors based on *F*^2^ are statistically about twice as large as those based on *F*, and *R*- factors based on ALL data will be even larger.

### Fractional atomic coordinates and isotropic or equivalent isotropic displacement parameters (Å^2^)

**Table:**

	*x*	*y*	*z*	*U*_iso_*/*U*_eq_	Occ. (<1)
Cl1A	0.57064 (17)	0.5279 (2)	0.01572 (14)	0.0392 (6)	
Cl2A	0.24394 (15)	0.23473 (19)	0.01947 (15)	0.0345 (5)	
Cl3A	0.36624 (16)	0.57334 (18)	0.29128 (14)	0.0359 (6)	
C1A	0.4591 (6)	0.5412 (6)	0.1481 (5)	0.0198 (18)	
C2A	0.4709 (6)	0.4823 (8)	0.0664 (5)	0.0243 (18)	
C3A	0.4067 (6)	0.3860 (7)	0.0270 (5)	0.0253 (19)	
H3A	0.4169	0.3436	−0.0272	0.030*	
C4A	0.3279 (5)	0.3533 (7)	0.0685 (5)	0.0211 (18)	
C5A	0.3104 (6)	0.4107 (7)	0.1489 (5)	0.0230 (19)	
H5A	0.2540	0.3874	0.1754	0.028*	
C6A	0.3784 (6)	0.5025 (7)	0.1882 (5)	0.024 (2)	
N7A	0.5286 (5)	0.6360 (6)	0.1898 (4)	0.0204 (16)	
C8A	0.6213 (6)	0.6166 (7)	0.2412 (5)	0.0212 (18)	
C9A	0.6617 (6)	0.7376 (7)	0.2642 (5)	0.0214 (18)	
C10A	0.5858 (6)	0.8265 (7)	0.2234 (5)	0.0213 (18)	
N11A	0.5045 (5)	0.7670 (5)	0.1782 (4)	0.0253 (17)	
N12A	0.6581 (5)	0.4979 (6)	0.2602 (5)	0.0293 (18)	
H12A	0.7170	0.4757	0.2924	0.044*	
H12B	0.6059	0.4439	0.2307	0.044*	
C13A	0.7543 (6)	0.7689 (7)	0.3268 (5)	0.0219 (18)	
O14A	0.7867 (4)	0.8752 (5)	0.3480 (4)	0.0295 (13)	
O15A	0.8009 (4)	0.6589 (5)	0.3602 (3)	0.0332 (14)	
C16A	0.8971 (6)	0.6727 (8)	0.4209 (6)	0.040 (2)	
H16A	0.9339	0.7481	0.4021	0.048*	0.55
H16B	0.9385	0.5942	0.4176	0.048*	0.55
H16E	0.9145	0.7656	0.4289	0.048*	0.45
H16F	0.9503	0.6295	0.3939	0.048*	0.45
C17A	0.8798 (15)	0.6913 (19)	0.5162 (13)	0.058 (5)	0.55
H17A	0.9446	0.7011	0.5575	0.087*	0.55
H17B	0.8442	0.6159	0.5346	0.087*	0.55
H17C	0.8390	0.7693	0.5190	0.087*	0.55
C17C	0.892 (2)	0.614 (2)	0.5099 (16)	0.061 (7)	0.45
H17G	0.9576	0.6248	0.5507	0.091*	0.45
H17H	0.8765	0.5220	0.5019	0.091*	0.45
H17I	0.8399	0.6575	0.5366	0.091*	0.45
C18A	0.5858 (6)	0.9701 (8)	0.2227 (5)	0.026 (2)	
C19A	0.6629 (6)	1.0401 (7)	0.1920 (5)	0.0244 (19)	
H19A	0.7186	0.9979	0.1739	0.029*	
C20A	0.6530 (7)	1.1752 (8)	0.1895 (5)	0.027 (2)	
H20A	0.7033	1.2239	0.1674	0.033*	
N21A	0.5786 (6)	1.2395 (6)	0.2158 (4)	0.0310 (18)	
C22A	0.5073 (7)	1.1693 (8)	0.2450 (6)	0.035 (2)	
H22A	0.4530	1.2137	0.2641	0.043*	
C23A	0.5086 (6)	1.0385 (8)	0.2487 (6)	0.030 (2)	
H23A	0.4556	0.9935	0.2695	0.036*	
Cl1B	−0.06420 (16)	0.0303 (2)	0.01822 (15)	0.0411 (6)	
Cl2B	0.26316 (15)	−0.26448 (18)	0.01878 (15)	0.0345 (5)	
Cl3B	0.25295 (17)	0.0682 (2)	0.29378 (16)	0.0446 (7)	
C1B	0.0994 (6)	0.0406 (7)	0.1511 (5)	0.0200 (18)	
C2B	0.0545 (5)	−0.0170 (7)	0.0693 (5)	0.0222 (18)	
C3B	0.1029 (6)	−0.1110 (7)	0.0272 (5)	0.026 (2)	
H3B	0.0713	−0.1502	−0.0284	0.031*	
C4B	0.2012 (6)	−0.1465 (7)	0.0700 (5)	0.0231 (19)	
C5B	0.2481 (6)	−0.0938 (7)	0.1505 (5)	0.026 (2)	
H5B	0.3139	−0.1201	0.1783	0.031*	
C6B	0.1961 (6)	−0.0005 (7)	0.1902 (5)	0.023 (2)	
N7B	0.0500 (5)	0.1350 (6)	0.1943 (4)	0.0226 (16)	
C8B	−0.0235 (6)	0.1154 (7)	0.2442 (5)	0.0234 (19)	
C9B	−0.0555 (5)	0.2384 (7)	0.2654 (5)	0.0194 (18)	
C10B	0.0031 (5)	0.3242 (7)	0.2249 (5)	0.0183 (18)	
N11B	0.0673 (4)	0.2660 (5)	0.1808 (4)	0.0218 (16)	
N12B	−0.0504 (5)	−0.0043 (6)	0.2651 (4)	0.0339 (19)	
H12C	−0.0959	−0.0024	0.3007	0.051*	
H12D	−0.0345	−0.0845	0.2525	0.051*	
C13B	−0.1254 (5)	0.2666 (7)	0.3257 (5)	0.0211 (18)	
O14B	−0.1505 (4)	0.3747 (4)	0.3467 (3)	0.0260 (13)	
O15B	−0.1612 (4)	0.1577 (4)	0.3584 (3)	0.0269 (12)	
C16B	−0.2347 (6)	0.1699 (8)	0.4178 (5)	0.030 (2)	
H16C	−0.2828	0.0967	0.4068	0.036*	
H16D	−0.2732	0.2513	0.4036	0.036*	
C17B	−0.1852 (7)	0.1706 (12)	0.5147 (6)	0.070 (3)	
H17D	−0.1473	0.0899	0.5289	0.105*	
H17E	−0.2363	0.1778	0.5535	0.105*	
H17F	−0.1390	0.2445	0.5260	0.105*	
C18B	0.0028 (6)	0.4676 (7)	0.2232 (5)	0.019 (2)	
C19B	−0.0840 (6)	0.5390 (8)	0.1929 (5)	0.0244 (19)	
H19B	−0.1467	0.4962	0.1742	0.029*	
C20B	−0.0789 (7)	0.6697 (8)	0.1900 (5)	0.036 (2)	
H20B	−0.1392	0.7162	0.1685	0.044*	
N21B	0.0062 (6)	0.7378 (7)	0.2158 (4)	0.0343 (18)	
C22B	0.0891 (7)	0.6710 (7)	0.2461 (5)	0.026 (2)	
H22B	0.1503	0.7165	0.2659	0.031*	
C23B	0.0897 (6)	0.5364 (7)	0.2499 (5)	0.0227 (19)	
H23B	0.1510	0.4919	0.2712	0.027*	
S1	0.5410 (2)	0.3311 (3)	0.5460 (2)	0.0470 (10)	0.737 (6)
S1A	0.5038 (7)	0.3249 (8)	0.4509 (6)	0.052 (3)	0.263 (6)
O4L	0.5551 (5)	0.1999 (6)	0.5035 (5)	0.082 (2)	
C2L	0.5902 (7)	0.4454 (8)	0.4825 (7)	0.071 (3)	
H2LA	0.5829	0.5327	0.5066	0.106*	0.737 (6)
H2LB	0.5547	0.4409	0.4189	0.106*	0.737 (6)
H2LC	0.6619	0.4264	0.4850	0.106*	0.737 (6)
H2LD	0.6482	0.4298	0.4526	0.106*	0.263 (6)
H2LE	0.6122	0.4438	0.5490	0.106*	0.263 (6)
H2LF	0.5609	0.5304	0.4638	0.106*	0.263 (6)
C3L	0.4116 (6)	0.3696 (9)	0.5097 (7)	0.071 (3)	
H3LA	0.3731	0.2897	0.4944	0.106*	0.737 (6)
H3LB	0.4039	0.4257	0.4558	0.106*	0.737 (6)
H3LC	0.3865	0.4147	0.5590	0.106*	0.737 (6)
H3LD	0.3658	0.2965	0.5121	0.106*	0.263 (6)
H3LE	0.3738	0.4430	0.4788	0.106*	0.263 (6)
H3LF	0.4423	0.3950	0.5720	0.106*	0.263 (6)

### Atomic displacement parameters (Å^2^)

**Table:**

	*U*^11^	*U*^22^	*U*^33^	*U*^12^	*U*^13^	*U*^23^
Cl1A	0.0387 (13)	0.0396 (13)	0.0439 (14)	−0.0159 (12)	0.0199 (12)	−0.0048 (10)
Cl2A	0.0275 (12)	0.0305 (12)	0.0457 (14)	−0.0121 (10)	0.0072 (11)	−0.0151 (10)
Cl3A	0.0334 (14)	0.0387 (12)	0.0377 (13)	−0.0066 (10)	0.0120 (11)	−0.0170 (9)
C1A	0.019 (5)	0.010 (4)	0.027 (5)	−0.004 (4)	−0.006 (4)	0.003 (3)
C2A	0.024 (5)	0.019 (4)	0.032 (4)	0.002 (4)	0.009 (4)	0.008 (3)
C3A	0.027 (5)	0.022 (4)	0.029 (5)	0.002 (4)	0.009 (4)	−0.001 (3)
C4A	0.013 (4)	0.014 (4)	0.037 (5)	−0.001 (3)	0.006 (4)	−0.004 (4)
C5A	0.018 (5)	0.022 (4)	0.030 (5)	−0.006 (4)	0.007 (4)	0.000 (3)
C6A	0.022 (5)	0.022 (5)	0.029 (5)	0.003 (4)	0.006 (4)	0.004 (4)
N7A	0.020 (4)	0.011 (4)	0.030 (4)	−0.005 (3)	0.003 (3)	−0.002 (3)
C8A	0.016 (5)	0.025 (4)	0.026 (5)	0.005 (4)	0.009 (4)	−0.004 (4)
C9A	0.021 (5)	0.017 (4)	0.025 (4)	0.007 (4)	0.003 (4)	0.002 (3)
C10A	0.024 (5)	0.013 (4)	0.027 (4)	−0.006 (4)	0.006 (4)	−0.005 (3)
N11A	0.025 (4)	0.009 (3)	0.041 (4)	0.001 (3)	0.001 (3)	−0.002 (3)
N12A	0.014 (4)	0.013 (4)	0.056 (5)	0.007 (3)	−0.005 (4)	0.003 (3)
C13A	0.021 (5)	0.027 (5)	0.018 (4)	−0.001 (4)	0.004 (4)	0.001 (3)
O14A	0.023 (3)	0.019 (3)	0.044 (4)	−0.010 (3)	0.001 (3)	−0.004 (3)
O15A	0.025 (3)	0.023 (3)	0.045 (3)	0.002 (3)	−0.011 (3)	0.002 (3)
C16A	0.018 (5)	0.042 (5)	0.056 (6)	−0.006 (4)	−0.005 (4)	0.004 (5)
C17A	0.053 (9)	0.068 (9)	0.051 (9)	−0.009 (7)	0.006 (7)	0.005 (7)
C17C	0.063 (10)	0.068 (10)	0.050 (9)	−0.009 (8)	0.007 (8)	0.008 (8)
C18A	0.027 (6)	0.018 (4)	0.029 (5)	0.000 (4)	−0.005 (4)	0.002 (4)
C19A	0.021 (5)	0.020 (4)	0.032 (5)	0.008 (4)	0.005 (4)	−0.005 (4)
C20A	0.030 (6)	0.024 (5)	0.024 (5)	−0.012 (4)	−0.007 (4)	0.007 (4)
N21A	0.033 (5)	0.016 (4)	0.039 (5)	0.000 (4)	−0.007 (4)	0.004 (3)
C22A	0.027 (6)	0.021 (5)	0.054 (6)	0.005 (4)	−0.005 (5)	0.005 (4)
C23A	0.025 (6)	0.021 (5)	0.043 (6)	0.000 (4)	0.004 (4)	−0.006 (4)
Cl1B	0.0287 (12)	0.0421 (14)	0.0483 (14)	0.0136 (12)	−0.0044 (12)	−0.0069 (11)
Cl2B	0.0289 (12)	0.0296 (12)	0.0467 (14)	0.0048 (11)	0.0115 (11)	−0.0152 (10)
Cl3B	0.0312 (14)	0.0560 (15)	0.0439 (15)	0.0066 (11)	−0.0003 (11)	−0.0287 (11)
C1B	0.012 (4)	0.018 (4)	0.032 (5)	−0.006 (3)	0.007 (4)	−0.001 (3)
C2B	0.016 (4)	0.021 (3)	0.030 (4)	0.010 (3)	0.004 (3)	0.002 (3)
C3B	0.028 (5)	0.022 (4)	0.031 (5)	0.003 (4)	0.013 (4)	−0.003 (3)
C4B	0.031 (5)	0.012 (4)	0.031 (5)	0.005 (4)	0.018 (4)	0.001 (3)
C5B	0.021 (5)	0.030 (5)	0.027 (5)	−0.003 (4)	0.007 (4)	−0.005 (3)
C6B	0.032 (6)	0.014 (4)	0.022 (5)	−0.010 (4)	0.004 (4)	−0.012 (3)
N7B	0.023 (4)	0.009 (3)	0.040 (4)	−0.007 (3)	0.017 (3)	−0.004 (3)
C8B	0.026 (5)	0.018 (4)	0.029 (5)	−0.003 (4)	0.012 (4)	0.000 (4)
C9B	0.009 (4)	0.016 (4)	0.034 (5)	0.009 (3)	0.005 (4)	−0.002 (4)
C10B	0.014 (5)	0.012 (4)	0.027 (5)	−0.005 (4)	0.001 (4)	−0.005 (3)
N11B	0.022 (4)	0.011 (3)	0.033 (4)	−0.006 (3)	0.007 (3)	−0.004 (3)
N12B	0.044 (5)	0.015 (4)	0.049 (5)	0.001 (3)	0.025 (4)	−0.003 (3)
C13B	0.016 (5)	0.017 (4)	0.029 (5)	−0.006 (3)	−0.001 (4)	−0.003 (3)
O14B	0.025 (3)	0.017 (3)	0.038 (3)	0.003 (2)	0.012 (3)	0.001 (2)
O15B	0.029 (3)	0.016 (3)	0.040 (3)	0.001 (2)	0.019 (3)	0.004 (2)
C16B	0.027 (5)	0.028 (4)	0.042 (5)	0.003 (4)	0.025 (4)	0.005 (4)
C17B	0.044 (6)	0.121 (10)	0.052 (7)	0.009 (6)	0.024 (5)	0.005 (7)
C18B	0.025 (6)	0.015 (4)	0.019 (5)	0.003 (4)	0.008 (4)	0.003 (3)
C19B	0.028 (5)	0.023 (4)	0.023 (5)	0.004 (4)	0.009 (4)	0.001 (4)
C20B	0.047 (7)	0.022 (5)	0.039 (6)	0.013 (5)	0.002 (5)	0.005 (4)
N21B	0.047 (5)	0.026 (4)	0.035 (5)	0.001 (4)	0.019 (4)	−0.001 (3)
C22B	0.029 (5)	0.019 (4)	0.032 (5)	−0.004 (4)	0.009 (4)	−0.011 (4)
C23B	0.024 (5)	0.010 (4)	0.038 (5)	0.004 (4)	0.016 (4)	0.001 (4)
S1	0.059 (2)	0.0423 (18)	0.043 (2)	0.0101 (15)	0.0180 (17)	0.0031 (14)
S1A	0.060 (6)	0.043 (5)	0.057 (7)	−0.005 (4)	0.025 (5)	−0.008 (4)
O4L	0.083 (5)	0.039 (4)	0.140 (7)	0.005 (4)	0.066 (5)	0.004 (4)
C2L	0.067 (7)	0.037 (5)	0.114 (9)	0.012 (5)	0.031 (7)	0.005 (5)
C3L	0.060 (6)	0.072 (7)	0.093 (8)	0.016 (6)	0.049 (6)	0.009 (6)

### Geometric parameters (Å, º)

**Table:**

Cl1A—C2A	1.724 (7)	C3B—C4B	1.412 (11)
Cl2A—C4A	1.736 (7)	C3B—H3B	0.9500
Cl3A—C6A	1.735 (8)	C4B—C5B	1.363 (9)
C1A—C6A	1.393 (10)	C5B—C6B	1.386 (10)
C1A—C2A	1.395 (10)	C5B—H5B	0.9500
C1A—N7A	1.419 (9)	N7B—C8B	1.358 (9)
C2A—C3A	1.378 (10)	N7B—N11B	1.394 (7)
C3A—C4A	1.365 (10)	C8B—N12B	1.340 (9)
C3A—H3A	0.9500	C8B—C9B	1.396 (9)
C4A—C5A	1.394 (9)	C9B—C10B	1.394 (9)
C5A—C6A	1.373 (10)	C9B—C13B	1.445 (9)
C5A—H5A	0.9500	C10B—N11B	1.324 (8)
N7A—C8A	1.357 (9)	C10B—C18B	1.480 (10)
N7A—N11A	1.394 (8)	N12B—H12C	0.8816
C8A—N12A	1.333 (9)	N12B—H12D	0.8840
C8A—C9A	1.381 (10)	C13B—O14B	1.223 (7)
C9A—C10A	1.426 (10)	C13B—O15B	1.349 (8)
C9A—C13A	1.454 (10)	O15B—C16B	1.450 (8)
C10A—N11A	1.330 (9)	C16B—C17B	1.475 (10)
C10A—C18A	1.482 (11)	C16B—H16C	0.9900
N12A—H12A	0.8811	C16B—H16D	0.9900
N12A—H12B	0.9422	C17B—H17D	0.9800
C13A—O14A	1.203 (8)	C17B—H17E	0.9800
C13A—O15A	1.349 (8)	C17B—H17F	0.9800
O15A—C16A	1.448 (9)	C18B—C23B	1.368 (10)
C16A—C17C	1.47 (2)	C18B—C19B	1.389 (10)
C16A—C17A	1.49 (2)	C19B—C20B	1.352 (11)
C16A—H16A	0.9900	C19B—H19B	0.9500
C16A—H16B	0.9900	C20B—N21B	1.342 (10)
C16A—H16E	0.9900	C20B—H20B	0.9500
C16A—H16F	0.9900	N21B—C22B	1.321 (9)
C17A—H17A	0.9800	C22B—C23B	1.390 (10)
C17A—H17B	0.9800	C22B—H22B	0.9500
C17A—H17C	0.9800	C23B—H23B	0.9500
C17C—H17G	0.9800	S1—O4L	1.521 (7)
C17C—H17H	0.9800	S1—C2L	1.720 (10)
C17C—H17I	0.9800	S1—C3L	1.777 (9)
C18A—C23A	1.372 (11)	S1—H2LE	1.5047
C18A—C19A	1.409 (10)	S1A—O4L	1.600 (11)
C19A—C20A	1.400 (11)	S1A—C3L	1.711 (12)
C19A—H19A	0.9500	S1A—C2L	1.711 (12)
C20A—N21A	1.321 (10)	S1A—H2LB	1.5006
C20A—H20A	0.9500	C2L—H2LA	0.9813
N21A—C22A	1.339 (10)	C2L—H2LB	0.9814
C22A—C23A	1.352 (11)	C2L—H2LC	0.9814
C22A—H22A	0.9500	C2L—H2LD	0.9818
C23A—H23A	0.9500	C2L—H2LE	0.9815
Cl1B—C2B	1.715 (7)	C2L—H2LF	0.9813
Cl2B—C4B	1.730 (7)	C3L—H3LA	0.9800
Cl3B—C6B	1.744 (7)	C3L—H3LB	0.9800
C1B—C2B	1.392 (10)	C3L—H3LC	0.9800
C1B—C6B	1.395 (10)	C3L—H3LD	0.9800
C1B—N7B	1.401 (9)	C3L—H3LE	0.9800
C2B—C3B	1.382 (9)	C3L—H3LF	0.9800
			
C6A—C1A—C2A	118.1 (7)	C8B—N7B—N11B	112.4 (6)
C6A—C1A—N7A	121.2 (7)	C8B—N7B—C1B	127.1 (6)
C2A—C1A—N7A	120.7 (7)	N11B—N7B—C1B	120.2 (6)
C3A—C2A—C1A	121.5 (7)	N12B—C8B—N7B	121.3 (7)
C3A—C2A—Cl1A	119.5 (6)	N12B—C8B—C9B	132.7 (8)
C1A—C2A—Cl1A	119.0 (6)	N7B—C8B—C9B	106.0 (6)
C4A—C3A—C2A	117.9 (7)	C10B—C9B—C8B	104.9 (6)
C4A—C3A—H3A	121.0	C10B—C9B—C13B	128.8 (7)
C2A—C3A—H3A	121.1	C8B—C9B—C13B	126.0 (7)
C3A—C4A—C5A	123.4 (7)	N11B—C10B—C9B	113.6 (6)
C3A—C4A—Cl2A	119.1 (6)	N11B—C10B—C18B	116.4 (7)
C5A—C4A—Cl2A	117.4 (6)	C9B—C10B—C18B	130.0 (7)
C6A—C5A—C4A	117.0 (7)	C10B—N11B—N7B	103.1 (6)
C6A—C5A—H5A	121.5	C8B—N12B—H12C	111.5
C4A—C5A—H5A	121.5	C8B—N12B—H12D	136.8
C5A—C6A—C1A	122.0 (7)	H12C—N12B—H12D	111.7
C5A—C6A—Cl3A	120.3 (6)	O14B—C13B—O15B	122.3 (7)
C1A—C6A—Cl3A	117.7 (6)	O14B—C13B—C9B	125.8 (7)
C8A—N7A—N11A	112.6 (6)	O15B—C13B—C9B	112.0 (6)
C8A—N7A—C1A	127.8 (6)	C13B—O15B—C16B	118.5 (6)
N11A—N7A—C1A	119.6 (6)	O15B—C16B—C17B	111.0 (6)
N12A—C8A—N7A	121.7 (7)	O15B—C16B—H16C	109.4
N12A—C8A—C9A	131.5 (8)	C17B—C16B—H16C	109.4
N7A—C8A—C9A	106.8 (6)	O15B—C16B—H16D	109.4
C8A—C9A—C10A	104.8 (7)	C17B—C16B—H16D	109.4
C8A—C9A—C13A	128.0 (7)	H16C—C16B—H16D	108.0
C10A—C9A—C13A	126.7 (7)	C16B—C17B—H17D	109.5
N11A—C10A—C9A	112.5 (7)	C16B—C17B—H17E	109.5
N11A—C10A—C18A	117.3 (7)	H17D—C17B—H17E	109.5
C9A—C10A—C18A	130.2 (7)	C16B—C17B—H17F	109.5
C10A—N11A—N7A	103.4 (6)	H17D—C17B—H17F	109.5
C8A—N12A—H12A	128.3	H17E—C17B—H17F	109.5
C8A—N12A—H12B	103.1	C23B—C18B—C19B	116.6 (7)
H12A—N12A—H12B	128.6	C23B—C18B—C10B	121.0 (7)
O14A—C13A—O15A	123.3 (7)	C19B—C18B—C10B	122.4 (7)
O14A—C13A—C9A	126.9 (7)	C20B—C19B—C18B	119.7 (8)
O15A—C13A—C9A	109.8 (6)	C20B—C19B—H19B	120.2
C13A—O15A—C16A	116.9 (6)	C18B—C19B—H19B	120.2
O15A—C16A—C17C	110.0 (12)	N21B—C20B—C19B	124.0 (8)
O15A—C16A—C17A	109.2 (10)	N21B—C20B—H20B	118.0
O15A—C16A—H16A	109.8	C19B—C20B—H20B	118.0
C17A—C16A—H16A	109.8	C22B—N21B—C20B	116.9 (7)
O15A—C16A—H16B	109.8	N21B—C22B—C23B	122.2 (8)
C17A—C16A—H16B	109.8	N21B—C22B—H22B	118.9
H16A—C16A—H16B	108.3	C23B—C22B—H22B	118.9
O15A—C16A—H16E	109.7	C18B—C23B—C22B	120.6 (7)
C17C—C16A—H16E	109.7	C18B—C23B—H23B	119.7
O15A—C16A—H16F	109.7	C22B—C23B—H23B	119.7
C17C—C16A—H16F	109.7	O4L—S1—C2L	107.0 (4)
H16E—C16A—H16F	108.2	O4L—S1—C3L	105.3 (5)
C16A—C17A—H17A	109.5	C2L—S1—C3L	97.8 (5)
C16A—C17A—H17B	109.5	O4L—S1—H2LE	125.0
H17A—C17A—H17B	109.5	C3L—S1—H2LE	115.1
C16A—C17A—H17C	109.5	O4L—S1A—C3L	104.9 (6)
H17A—C17A—H17C	109.5	O4L—S1A—C2L	103.9 (6)
H17B—C17A—H17C	109.5	C3L—S1A—C2L	100.8 (7)
C16A—C17C—H17G	109.5	O4L—S1A—H2LB	128.1
C16A—C17C—H17H	109.5	C3L—S1A—H2LB	111.4
H17G—C17C—H17H	109.5	S1A—C2L—H2LA	131.2
C16A—C17C—H17I	109.5	S1—C2L—H2LA	110.7
H17G—C17C—H17I	109.5	S1—C2L—H2LB	109.1
H17H—C17C—H17I	109.5	H2LA—C2L—H2LB	109.5
C23A—C18A—C19A	118.1 (7)	S1A—C2L—H2LC	118.9
C23A—C18A—C10A	120.8 (8)	S1—C2L—H2LC	108.6
C19A—C18A—C10A	121.0 (8)	H2LA—C2L—H2LC	109.5
C20A—C19A—C18A	116.6 (8)	H2LB—C2L—H2LC	109.4
C20A—C19A—H19A	121.7	S1A—C2L—H2LD	108.4
C18A—C19A—H19A	121.7	S1—C2L—H2LD	123.8
N21A—C20A—C19A	124.5 (8)	H2LA—C2L—H2LD	117.3
N21A—C20A—H20A	117.8	S1A—C2L—H2LE	109.3
C19A—C20A—H20A	117.8	H2LB—C2L—H2LE	168.0
C20A—N21A—C22A	117.0 (7)	H2LD—C2L—H2LE	109.5
N21A—C22A—C23A	123.3 (8)	S1A—C2L—H2LF	110.7
N21A—C22A—H22A	118.4	S1—C2L—H2LF	126.4
C23A—C22A—H22A	118.4	H2LC—C2L—H2LF	122.3
C22A—C23A—C18A	120.5 (8)	H2LD—C2L—H2LF	109.5
C22A—C23A—H23A	119.7	H2LE—C2L—H2LF	109.5
C18A—C23A—H23A	119.7	S1—C3L—H3LA	109.5
C2B—C1B—C6B	117.4 (7)	S1—C3L—H3LB	109.5
C2B—C1B—N7B	122.0 (7)	H3LA—C3L—H3LB	109.5
C6B—C1B—N7B	120.5 (7)	S1A—C3L—H3LC	153.4
C3B—C2B—C1B	122.0 (7)	S1—C3L—H3LC	109.5
C3B—C2B—Cl1B	118.7 (6)	H3LA—C3L—H3LC	109.5
C1B—C2B—Cl1B	119.3 (6)	H3LB—C3L—H3LC	109.5
C2B—C3B—C4B	117.5 (7)	S1A—C3L—H3LD	109.5
C2B—C3B—H3B	121.3	S1—C3L—H3LD	114.1
C4B—C3B—H3B	121.3	H3LB—C3L—H3LD	120.7
C5B—C4B—C3B	122.8 (7)	S1A—C3L—H3LE	109.5
C5B—C4B—Cl2B	118.9 (6)	S1—C3L—H3LE	135.6
C3B—C4B—Cl2B	118.3 (6)	H3LA—C3L—H3LE	109.4
C4B—C5B—C6B	117.5 (7)	H3LD—C3L—H3LE	109.5
C4B—C5B—H5B	121.2	S1A—C3L—H3LF	109.5
C6B—C5B—H5B	121.2	H3LA—C3L—H3LF	124.1
C5B—C6B—C1B	122.8 (7)	H3LB—C3L—H3LF	125.5
C5B—C6B—Cl3B	118.9 (6)	H3LD—C3L—H3LF	109.5
C1B—C6B—Cl3B	118.3 (6)	H3LE—C3L—H3LF	109.5
			
C6A—C1A—C2A—C3A	−1.8 (11)	C2B—C3B—C4B—C5B	1.1 (11)
N7A—C1A—C2A—C3A	177.1 (7)	C2B—C3B—C4B—Cl2B	179.8 (5)
C6A—C1A—C2A—Cl1A	−179.6 (5)	C3B—C4B—C5B—C6B	−0.8 (12)
N7A—C1A—C2A—Cl1A	−0.7 (10)	Cl2B—C4B—C5B—C6B	−179.4 (6)
C1A—C2A—C3A—C4A	2.9 (11)	C4B—C5B—C6B—C1B	−0.1 (12)
Cl1A—C2A—C3A—C4A	−179.3 (6)	C4B—C5B—C6B—Cl3B	179.4 (6)
C2A—C3A—C4A—C5A	−1.2 (12)	C2B—C1B—C6B—C5B	0.6 (11)
C2A—C3A—C4A—Cl2A	178.7 (5)	N7B—C1B—C6B—C5B	179.8 (7)
C3A—C4A—C5A—C6A	−1.5 (12)	C2B—C1B—C6B—Cl3B	−178.9 (6)
Cl2A—C4A—C5A—C6A	178.7 (6)	N7B—C1B—C6B—Cl3B	0.3 (10)
C4A—C5A—C6A—C1A	2.6 (11)	C2B—C1B—N7B—C8B	77.2 (10)
C4A—C5A—C6A—Cl3A	−176.8 (6)	C6B—C1B—N7B—C8B	−101.9 (9)
C2A—C1A—C6A—C5A	−1.0 (11)	C2B—C1B—N7B—N11B	−96.2 (8)
N7A—C1A—C6A—C5A	−179.9 (7)	C6B—C1B—N7B—N11B	84.7 (9)
C2A—C1A—C6A—Cl3A	178.4 (6)	N11B—N7B—C8B—N12B	−179.4 (7)
N7A—C1A—C6A—Cl3A	−0.5 (10)	C1B—N7B—C8B—N12B	6.7 (12)
C6A—C1A—N7A—C8A	99.6 (9)	N11B—N7B—C8B—C9B	−0.6 (9)
C2A—C1A—N7A—C8A	−79.3 (10)	C1B—N7B—C8B—C9B	−174.4 (7)
C6A—C1A—N7A—N11A	−81.7 (9)	N12B—C8B—C9B—C10B	178.8 (9)
C2A—C1A—N7A—N11A	99.4 (8)	N7B—C8B—C9B—C10B	0.2 (8)
N11A—N7A—C8A—N12A	179.9 (7)	N12B—C8B—C9B—C13B	5.5 (14)
C1A—N7A—C8A—N12A	−1.3 (12)	N7B—C8B—C9B—C13B	−173.1 (7)
N11A—N7A—C8A—C9A	−0.6 (9)	C8B—C9B—C10B—N11B	0.3 (8)
C1A—N7A—C8A—C9A	178.2 (7)	C13B—C9B—C10B—N11B	173.3 (7)
N12A—C8A—C9A—C10A	179.8 (8)	C8B—C9B—C10B—C18B	179.4 (7)
N7A—C8A—C9A—C10A	0.4 (8)	C13B—C9B—C10B—C18B	−7.6 (12)
N12A—C8A—C9A—C13A	−8.1 (14)	C9B—C10B—N11B—N7B	−0.6 (8)
N7A—C8A—C9A—C13A	172.5 (7)	C18B—C10B—N11B—N7B	−179.8 (6)
C8A—C9A—C10A—N11A	−0.1 (9)	C8B—N7B—N11B—C10B	0.7 (8)
C13A—C9A—C10A—N11A	−172.3 (7)	C1B—N7B—N11B—C10B	175.1 (6)
C8A—C9A—C10A—C18A	−178.8 (8)	C10B—C9B—C13B—O14B	5.5 (12)
C13A—C9A—C10A—C18A	9.0 (13)	C8B—C9B—C13B—O14B	177.2 (8)
C9A—C10A—N11A—N7A	−0.3 (8)	C10B—C9B—C13B—O15B	−174.0 (7)
C18A—C10A—N11A—N7A	178.6 (6)	C8B—C9B—C13B—O15B	−2.3 (10)
C8A—N7A—N11A—C10A	0.5 (8)	O14B—C13B—O15B—C16B	2.3 (10)
C1A—N7A—N11A—C10A	−178.4 (6)	C9B—C13B—O15B—C16B	−178.2 (6)
C8A—C9A—C13A—O14A	−177.7 (8)	C13B—O15B—C16B—C17B	−93.4 (9)
C10A—C9A—C13A—O14A	−7.2 (13)	N11B—C10B—C18B—C23B	−53.7 (10)
C8A—C9A—C13A—O15A	1.7 (11)	C9B—C10B—C18B—C23B	127.2 (9)
C10A—C9A—C13A—O15A	172.2 (7)	N11B—C10B—C18B—C19B	124.8 (8)
O14A—C13A—O15A—C16A	−3.2 (11)	C9B—C10B—C18B—C19B	−54.3 (11)
C9A—C13A—O15A—C16A	177.4 (6)	C23B—C18B—C19B—C20B	0.8 (12)
C13A—O15A—C16A—C17C	120.8 (13)	C10B—C18B—C19B—C20B	−177.8 (7)
C13A—O15A—C16A—C17A	86.6 (11)	C18B—C19B—C20B—N21B	−0.5 (13)
N11A—C10A—C18A—C23A	53.4 (10)	C19B—C20B—N21B—C22B	−0.4 (12)
C9A—C10A—C18A—C23A	−128.0 (10)	C20B—N21B—C22B—C23B	1.2 (11)
N11A—C10A—C18A—C19A	−124.0 (8)	C19B—C18B—C23B—C22B	−0.1 (11)
C9A—C10A—C18A—C19A	54.6 (11)	C10B—C18B—C23B—C22B	178.5 (6)
C23A—C18A—C19A—C20A	−0.8 (12)	N21B—C22B—C23B—C18B	−1.0 (12)
C10A—C18A—C19A—C20A	176.7 (6)	C2L—S1—O4L—S1A	−52.1 (5)
C18A—C19A—C20A—N21A	1.7 (12)	C3L—S1—O4L—S1A	51.3 (5)
C19A—C20A—N21A—C22A	−1.4 (11)	C3L—S1A—O4L—S1	−54.0 (5)
C20A—N21A—C22A—C23A	0.2 (12)	C2L—S1A—O4L—S1	51.3 (5)
N21A—C22A—C23A—C18A	0.6 (13)	O4L—S1A—C2L—S1	−47.9 (5)
C19A—C18A—C23A—C22A	−0.2 (13)	C3L—S1A—C2L—S1	60.5 (5)
C10A—C18A—C23A—C22A	−177.7 (7)	O4L—S1—C2L—S1A	52.5 (5)
C6B—C1B—C2B—C3B	−0.2 (11)	C3L—S1—C2L—S1A	−56.2 (6)
N7B—C1B—C2B—C3B	−179.4 (7)	O4L—S1A—C3L—S1	49.0 (5)
C6B—C1B—C2B—Cl1B	−179.8 (6)	C2L—S1A—C3L—S1	−58.7 (5)
N7B—C1B—C2B—Cl1B	1.1 (10)	O4L—S1—C3L—S1A	−52.7 (5)
C1B—C2B—C3B—C4B	−0.6 (11)	C2L—S1—C3L—S1A	57.5 (6)
Cl1B—C2B—C3B—C4B	179.0 (6)		

### Hydrogen-bond geometry (Å, º)

**Table:**

*D*—H···*A*	*D*—H	H···*A*	*D*···*A*	*D*—H···*A*
N12*A*—H12*A*···O15*A*	0.88	2.34	2.767 (8)	110
N12*A*—H12*A*···O14*B*^i^	0.88	2.10	2.957 (8)	164
N12*A*—H12*B*···N21*A*^ii^	0.94	2.15	2.906 (9)	137
N12*B*—H12*C*···O15*B*	0.88	2.13	2.778 (8)	130
N12*B*—H12*C*···O14*A*^iii^	0.88	2.24	2.983 (8)	142
N12*B*—H12*D*···N21*B*^ii^	0.88	2.02	2.901 (9)	176

Symmetry codes: (i) *x*+1, *y*, *z*; (ii) *x*, *y*−1, *z*; (iii) *x*−1, *y*−1, *z*.
